# Correlation of sonographic and radiographic scores of lung edema and metrics of shunt, dead space, and respiratory mechanics in invasively ventilated patients

**DOI:** 10.62675/2965-2774.20250036

**Published:** 2025-09-25

**Authors:** Daan Filippini, Claudio Zimatore, Laura A. Hagens, Nanon F. L. Heijnen, Leila Atmowihardjo, Ronny M. Schnabel, Dennis C. J. J. Bergmans, Daniele Guerino Biasucci, Marcus J. Schultz, Lieuwe D. J. Bos, Marry R. Smit, Luigi Pisani

**Affiliations:** 1 Department of Intensive Care Medicine Amsterdam University Medical Centers Amsterdam The Netherlands Department of Intensive Care Medicine, Amsterdam University Medical Centers, Location “AMC” - Amsterdam, The Netherlands.; 2 Intensive Care Maastricht University Medical Center+ Maastricht University Maastricht The Netherlands Intensive Care, Maastricht University Medical Center+, Maastricht University -Maastricht, The Netherlands.; 3 Department of Clinical Science and Translational Medicine “Tor Vergata” University of Rome Rome Italy Department of Clinical Science and Translational Medicine, “Tor Vergata” University of Rome - Rome, Italy.; 4 Department of Precision and Regenerative Medicine and Ionian Area Division of Intensive Care University of Bari “Aldo Moro” Bari Italy Department of Precision and Regenerative Medicine and Ionian Area, Division of Intensive Care, University of Bari “Aldo Moro” - Bari, Italy.

**Keywords:** Lung, Ultrasonography, RALE score, Pulmonary edema, Respiratory dead space, Respiratory mechanics, Respiration, artificial, Ventilatory ratio, Mechanical power, Diagnostic imaging

## Abstract

**Objective:**

To investigate the relationship between sonographic and radiological scores of lung edema with metrics of shunt, dead space, and respiratory mechanics in critically ill patients under invasive ventilation for greater than 24 hours.

**Methods:**

This is a secondary analysis of a prospective observational study involving invasively ventilated critically ill patients. The radiographic assessment of lung edema score and the global lung ultrasound score were utilized to evaluate pulmonary edema. Measurements for assessing shunt and dead space included the ratio of partial pressure of oxygen to fraction of inspired oxygen ratio, ventilatory ratio, and corrected minute volume, respectively. Respiratory mechanics were assessed through dynamic respiratory system compliance, driving pressure, and mechanical power of ventilation.

**Results:**

A total of 364 invasively ventilated patients were included; one-third of them were classified as having acute respiratory distress syndrome. Median radiographic assessment of lung edema and global lung ultrasound scores were 15 [8 to 20] and 7 [3 to 13], respectively. Both scores explained little of the variance in partial pressure of oxygen to fraction of inspired oxygen ratio, ventilatory ratio, corrected minute volume, respiratory system compliance, driving pressure, and mechanical power (R^2^ = 0.05-0.12). Patients without acute respiratory distress syndrome exhibited a stronger association between the radiographic assessment of lung edema score and partial pressure of oxygen to fraction of inspired oxygen ratio, as well as between the global lung ultrasound score and respiratory system compliance. In contrast, patients with acute respiratory distress syndrome demonstrated stronger associations between the radiographic assessment of lung edema score and mechanical power and between the global lung ultrasound score and dead space metrics. A positive interaction of positive end-expiratory pressure was found only for the association between partial pressure of oxygen to fraction of inspired oxygen ratio and the radiographic assessment of lung edema and global lung ultrasound scores.

**Conclusion:**

The radiographic assessment of lung edema score and the global lung ultrasound score poorly correlate with shunt, dead space, and respiratory mechanics metrics in invasively ventilated patients. A counterintuitive moderation effect of acute respiratory distress syndrome status is observed in some of these associations.

## INTRODUCTION

Assessing pulmonary aeration in invasively ventilated patients is crucial for understanding the distribution of ventilation, minimizing the risk of ventilator-induced lung injury, achieving optimal lung recruitment, preventing atelectasis, and ensuring effective gas exchange.^( 1 , 2 )^A subset of ventilated patients is admitted with or develops acute respiratory distress syndrome (ARDS). Independent of the presence of ARDS, the impairment of lung function may cause shunt, dead space, and abnormalities in pulmonary mechanics.

Atelectasis, contusions, consolidations, infarctions, edema, or pneumonitis in the lung cause increased shunt. Increased dead-space is a marker of ARDS severity and a predictor of survival, independent of oxygenation. Intravascular thrombi, endothelial injury, heterogeneity of the ventilation-to-perfusion ratio, overdistension of alveolar units, and low cardiac output contribute to increased dead space fraction.^( 3 )^Common physiological metrics are used as surrogate measures for pulmonary shunt and dead space, substituting the more cumbersome direct measurements.^( 4 - 6 )^ Respiratory mechanics metrics include respiratory system compliance (C_RS_) and driving pressure (ΔP), which have been proposed as bedside measures to titrate mechanical ventilation settings and, in particular, positive end-expiratory pressure (PEEP).^( 7 )^Mechanical power (MP) is a calculated metric that captures the amount of energy transferred from the ventilator to the respiratory system, increasingly proposed as a ventilation target to improve care of critically ill patients.^( 8 )^

Lung ultrasound (LUS) aeration score and the radiographic assessment of lung edema (RALE) score are increasingly used to estimate pulmonary aeration, monitor lung edema progression, and the onset of complications.^( 9 - 12 )^ While the relationship between these morphological scores and biomarkers was recently evaluated in the Diagnosis of Acute Respiratory Distress Syndrome (DARTS) cohort,^( 13 )^the relationship with physiological metrics of shunt, dead space, and compliance is still scarcely investigated.^( 14 )^ In the present analysis, we thus aim to investigate the relationship between sonographic and radiological scores of lung edema with metrics of shunt, dead space, and respiratory mechanics in critically ill patients under invasive ventilation for greater than 24 hours. We hypothesized that there is a notable association between the RALE score and LUS score, and metrics of shunt, dead space, and various measures of respiratory mechanics, and that this association would be stronger in ARDS compared to those without ARDS.

## METHODS

### Study design and ethics

This is a secondary analysis of the DARTS project.^( 15 )^The DARTS project is a prospective observational study that included invasively ventilated patients in two tertiary hospitals in the Netherlands (Amsterdam University Medical Center [UMC], location AMC, and Maastricht University Medical Center). Patients were consecutively included from 03-2019 until 03-2021. The Amsterdam UMC institutional review board approved the trial (W18_311 and 2018_287), and written deferred consent for the use of data was obtained from patients and/or their legal representatives. The LUS examination was performed within 48 hours of intubation, and all data were collected at this moment. A chest X-ray was only included if available in the 24 hours preceding the LUS examination.

### Patients

The DARTS study included patients admitted to the intensive care unit (ICU) and expected to be invasively ventilated for at least 24 hours. Exclusion criteria for participation in DARTS included invasive ventilation for more than 48 hours in the week before inclusion, moribund patients, tracheotomised patients, if breath sampling was deemed clinically inappropriate, and refusal of patients or their legal representatives to participate in the study.

For this secondary analysis, the unavailability of either baseline chest X-rays or LUS was an additional exclusion criterion.

### Radiographic assessment of lung edema scoring

The RALE score is calculated by first dividing the lungs into four quadrants. This division is achieved horizontally by a line through the first branch of the left main bronchus and vertically by the spinal column. Within these quadrants, the degree of consolidation is rated on a scale from zero to 4 (0% = 0, < 25% = 1, 25 - 50% = 2, 50 - 75% = 3, > 75% = 4), and the density is scored from 1 to 3 (1 = hazy, 2 = moderate, 3 = dense). The scores for consolidation extent and density are multiplied in each quadrant, and the total RALE score, which can range from zero to 48, is the sum of these quadrant scores.

The quality of all chest X-rays was initially evaluated by one reviewer, who categorized them as acceptable, borderline, or unusable, considering overall quality and potential confounding factors like pleural effusion, pneumothorax, or subcutaneous emphysema. Chest X-rays deemed acceptable were scored by the same reviewer, with inter-observer agreement verified through a 10% random sample evaluated by a second reviewer. Chest X-rays labelled as borderline or unusable underwent assessment and scoring by a team of three reviewers in a consensus meeting. All reviewers received training from an expert until they achieved an intraclass correlation coefficient (ICC) of over 0.9 with the expert. Importantly, they were blinded to all other patient data during the review process.

There was good inter-observer agreement between the RALE scores (ICC = 0.78, 95% confidence interval [95%CI]: 0.65 - 0.87). Of the 422 baseline chest X-rays, 40 with doubtful quality were scored together in a consensus meeting, and 5 were deemed unusable.

### Lung ultrasound examination

The LUS exam consisted of 12 scanned regions, with each hemithorax divided into two anterior, two lateral, and two posterior regions. A linear probe was used in a transverse scanning position. An A-pattern was characterized by horizontal repetitions of the pleural line (A-lines) and scored as zero. B-patterns were scored as 1 if more than two well-spaced B-lines were present, covering less than 50% of the pleural line, or 2 if B-lines covered more than 50% of the pleural line. A C-pattern denoted consolidation or near complete loss of aeration larger than 2cm, scored as 3. C-patterns with pleural effusion were scored as zero, suggesting compression atelectasis rather than intrinsic pulmonary pathology;^( 16 )^however, of note, the standard operating procedure only allowed for assigning a pleural effusion present if > 2cm, mitigating any over-scoring of clinically irrelevant effusions. Each LUS image was scored according to the worst pattern observed, resulting in the LUS aeration score for the individual image. The global LUS aeration score (range zero to 36) was calculated as the sum of the LUS aeration scores of all regions.^( 17 )^Global LUS aeration scores were considered eligible if the examination was done on the same day as the radiography. Lung ultrasound examiners were trained by an expert physician beforehand, which has been shown to lead to an excellent interrater agreement (ICC = 0.98).^( 18 )^

### Endpoints

To assess shunt, the partial pressure of oxygen to fraction of inspired oxygen ratio (PaO_2_/FiO_2_) was used. For dead space, the ventilatory ratio and corrected minute volume were used. Respiratory mechanics was evaluated using dynamic C_RS_, ΔP, and MP. All endpoints were additionally tested for a possible moderating effect of PEEP.

### Definitions

Acute respiratory distress syndrome was diagnosed by an expert panel of three independent physicians using the Berlin criteria. A detailed description of this process is provided elsewhere.^( 15 )^ The following formulas were used for ventilatory ratio (minute volume * PaCO_2_)/ideal body weight * 100 * 37.5); corrected minute volume ((minute volume * PaCO_2_)/40), dynamic C_RS_ (tidal volume/(Pmax-PEEP)), ΔP (Pplat - PEEP) and MP (0.098 * respiratory rate * tidal volume * Pmax). In all calculations of respiratory mechanics metrics, Pmax was equal to the maximum airway pressure (Pmax) in pressure-controlled ventilation or peak pressure (Ppeak) in volume-controlled ventilation.

### Statistical analysis

Categorical data were expressed as numbers and percentages, and differences were tested using the Chi-squared test. Continuous data were expressed as median ± interquartile range [IQR] and differences were analysed depending on the parametric or non-parametric distribution using a t-test or one-way analysis of variance (ANOVA; parametric distribution), or a Mann-Whitney U or Kruskal-Wallis test (non-parametric distribution). The correlation between RALE scores and global LUS scores was displayed in a scatterplot and tested using the Pearson correlation coefficient. All outcomes were displayed using scatterplots and tested using linear regression. The statistical significance was determined by the p value. The explanatory power of the model was determined by the determination coefficient (R^2^), which refers to the explained variance of the dependent variables (physiological measures) upon changes in the independent variables (RALE or LUS scores). In the absence of predefined accepted definitions for the strength of associations, in the current analysis, we deemed an R^2^> 0.5 as a marker of a strong association, an R^2^between 0.2 and 0.5 as a moderate association, and an R^2^< 0.2 as a poor association.

The effect size was determined by reporting each association’s individual β-coefficients. In the linear regression, ARDS status and PEEP were added as an interaction term to test for possible moderating effects. Tests were two-sided with a significance level of 0.05. Data analysis was performed using R version 4.0.3 using the R studio interface.

## RESULTS

### Patients

**Figure 1:**
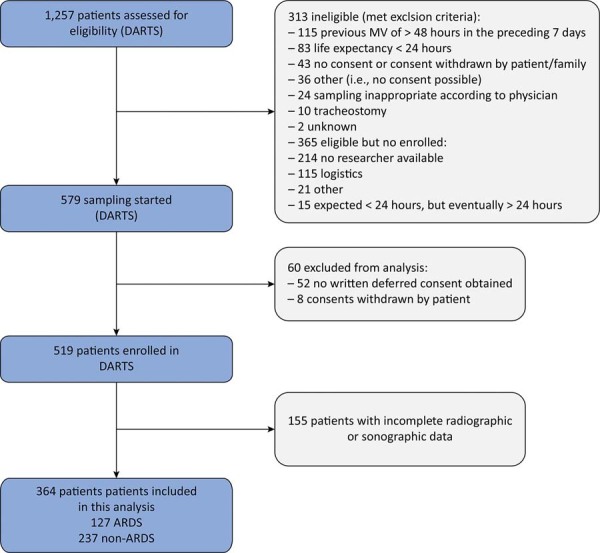
- Flowchart of patient inclusion.

This analysis included 364 out of 519 DARTS patients. The main reason for exclusion was not having both a chest X-ray and LUS available at baseline ( Figure 1 ). Most patients were male and admitted for a medical reason ( Table 1 ). One third (34.9%) of patients had ARDS according to the Berlin definition of ARDS, and 32.3% of the patients died in the ICU. Most patients were ventilated in pressure control or pressure support mode, with ARDS patients having a higher PEEP, ventilatory ratio, ΔP, and MP as compared to patients without ARDS ( Table 1 ).

DARTS - Diagnosis of Acute Respiratory Distress Syndrome; MV - mechanical ventilation; ARDS - acute respiratory distress syndrome.

**Table 1:** Patients’ demographic and clinical characteristics

	Overall	Non-ARDS	ARDS
	(n = 364)	(n = 237)	(n = 127)
Demographics			
Age (mean (SD))	62 ± 14	62 ± 15	63 ± 13
Male (%)	243 (67)	155 (65)	88 (69)
BMI (kg×m^2^)	26 [23 - 29]	26 [23 - 30]	26 [24 - 30]
Admission			
Hospital stay before ICU (days)	1 [0 - 1]	1 [0 - 1]	1 [0 - 2]
Emergency surgical	51 (14.0)	40 (16.9)	11 (8.7)
Medical	267 (73.4)	160 (67.5)	107 (84.3)
Planned surgical	46 (12.6)	37 (15.6)	9 (7.1)
Pneumonia (%)	115 (31.6)	29 (12.2)	86 (67.7)
COVID-19 (%)	37 (10.2)	1 (0.4)	36 (28.3)
Imaging scores			
RALE score	15 [8 - 20]	11 [7 - 17]	20 [15 - 28]
LUS score	7 [3 - 13]	5 [2 - 9]	12 [8 - 16]
Ventilation			
Ventilation mode			
Pressure control	145 (39.8)	94 (39.6)	51 (40.2)
Pressure support	122 (33.5)	74 (31.2)	48 (37.8)
Volume control	2 (0.5)	1 (0.4)	1 (0.8)
Intellivent	95 (26.1)	68 (28.7)	27 (21.3)
Pmax (cmH_2_O)	21 [16 - 26]	19 [15 - 24]	24 [18 - 29]
PEEP (cmH_2_O)	8 [5 - 10]	7 [5 - 8]	10 [8 - 12]
Ventilatory ratio	1.35 [1.10 - 1.74]	1.28 [1.01 - 1.58]	1.60 [1.22 - 1.94]
Corrected minute volume (L/minute)	8.1 [6.5 - 10.6]	7.6 [6.2 - 9.7]	9.2 [7.4 12.0]
Compliance (mL/cmH_2_O)	33 [24 - 50]	35 [25 - 51]	31 [23 - 47]
Driving pressure (cmH_2_O)	13 [9 - 18]	13 [9 - 17]	15 [9 - 19]
Mechanical power (Joules/minute)	17.9 [12.1 - 24.5]	15.3 [10.9 - 21.7]	22.0 [16.0 - 29.4]
Severity			
APACHE II score	20 [15 - 25]	20 [15 - 26]	20 [14.50 - 24]
SOFA score	9 [7 - 11]	9 [7 - 11]	9 [7 - 11]
PaO_2_/FiO_2_ (mmHg)	188 [119 - 280]	236 [157 - 326]	120 [88 - 170]
Lactate (mmol/L)	1.7 [1.2 - 2.5]	1.6 [1.1 - 2.5]	1.9 [1.4 - 2.5]
Outcomes			
ICU length of stay (days)	7 [3 - 13]	6 [3 - 11]	9 [5 - 16]
ICU mortality (%)	117 (32.3)	76 (32.3)	41 (32.3)

ARDS - acute respiratory distress syndrome; BMI - body mass index; ICU - intensive care unit; COVID-19 - coronavirus disease 2019; RALE - radiographic assessment of lung edema; LUS - lung ultrasound; Pmax - maximum pressure; PEEP - positive end-expiratory pressure; APACHE II - Acute Physiology and Chronic Health Evaluation II; SOFA - Sequential Organ Failure Assessment; PaO_2_/FiO_2 -_ partial pressure of oxygen to fraction of inspired oxygen ratio. Data expressed as mean (standard deviation), n (%) or median [interquartile range].

RALE - radiographic assessment of lung edema; PaO_2_/FiO_2 -_ partial pressure of oxygen to fraction of inspired oxygen ratio; LUS - lung ultrasound.

The line represents linear regression with the 95% confidence interval plotted around it in grey.

### Radiographic assessment of lung edema and lung ultrasound scores

The median RALE score was 15 [IQR = 8-20], while the median global LUS score was 7 [IQR = 3-13] ( Table 1 ). There was a weak correlation between the RALE and global LUS scores (r = 0.44; p < 0.001) ( Figure 1S - Supplementary Material ).

### Associations between radiographic assessment of lung edema and lung ultrasound scores and PaO2/FiO2

**Figure 2:**
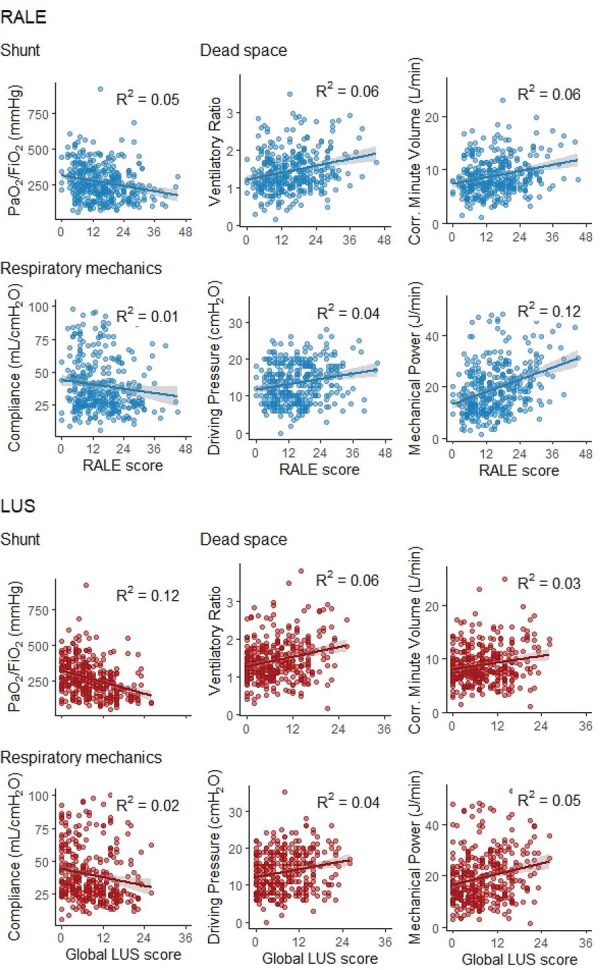
Association between the radiographic assessment of lung edema score and global lung ultrasound score with shunt, dead space, and respiratory mechanics metrics.

Both the RALE and the global LUS scores had a significant inverse association with PaO_2_/FiO_2_, but the scores alone explain minimal variance in oxygenation ( Figure 2 and Table 2 ). There is a moderation of ARDS status ( Table 1S - Supplementary Material ), and the association between RALE and PaO_2_/FiO_2_ is wholly lost in patients *with* ARDS ( Table 2S - Supplementary Material ), while it is weakly maintained in patients *without* ARDS ( Table 3S - Supplementary Material ).

**Table 2:** Linear regression outputs of baseline radiographic assessment of lung edema scores and baseline global lung ultrasound scores

	RALE score				LUS score			
	β	95%CI	p value	Adj. R^2^	β	95%CI	p value	Adj. R^2^
Shunt								
PaO_2_/FiO_2_								
Intercept	315	290 - 340	< 0.001	0.05	323	304 - 343	< 0.001	0.12
Score	-3.1	-4.5 - -1.7	< 0.001		-6.7	-8.6 - -4.8	< 0.001	
Dead space								
Ventilatory ratio								
Intercept	1.2	1.1 - 1.3	< 0.001	0.06	1.3	1.2 - 1.4	< 0.001	0.06
Score	0.02	0.01 - 0.02	< 0.001		0.02	0.01 - 0.03	< 0.001	
Corrected VE								
Intercept	7.3	6.6 - 8.0	< 0.001	0.06	8.0	7.4 - 8.6	< 0.001	0.03
Score	0.1	0.06 - 0.14	< 0.001		0.1	0.05 - 0.16	< 0.001	
Mechanics								
Compliance								
Intercept	43.5	39.2 - 47.8	< 0.001	0.01	43.7	40.1 - 47.2	< 0.001	0.02
Score	-0.28	-0.52 - -0.03	0.027		-0.53	-0.88 - -0.19	0.002	
Driving pressure								
Intercept	11.0	10.6 - 12.8	< 0.001	0.04	12.1	11.2 - 13.0	< 0.001	0.04
Score	0.12	0.06 - 0.18	< 0.001		0.18	0.09 - 0.26	< 0.001	
Mechanical power								
Intercept	13.3	11.4 - 15.2	< 0.001	0.12	16.5	14.9 - 18.1	< 0.001	0.05
Score	0.40	0.29 - 0.50	< 0.001		0.35	0.19 - 0.51	< 0.001	

RALE - radiographic assessment of lung edema; LUS - lung ultrasound; 95%CI - 95% confidence interval; PaO_2_/FiO_2 -_ partial pressure of oxygen to fraction of inspired oxygen ratio; VE - minute volume.

### Associations between radiographic assessment of lung edema and lung ultrasound scores and dead space

Both scores were poorly associated with ventilatory ratio and corrected minute volume. ARDS status is moderated only for the global LUS score ( Table 1S - Supplementary Material ). The association between the global LUS score and both dead space metrics is present in patients with ARDS ( Table 2S - Supplementary Material ), while it is lost in patients without ARDS ( Table 3S - Supplementary Material ).

### Association between radiographic assessment of lung edema and lung ultrasound scores and respiratory mechanics

Both RALE score and global LUS score were weakly associated with C_RS_, ΔP, and MP ( Table 2 ).

There is a moderation of ARDS status for the association between RALE and MP ( Table 1S - Supplementary Material ). The association is stronger in patients with ARDS as compared to patients without this condition ( Tables 2S and 3S - Supplementary Material ).

There is a moderation of ARDS status for the association between global LUS score and compliance ( Table 1S - Supplementary Material ). The association is lost in patients with ARDS ( Table 2S - Supplementary Material ), while maintained in patients without this condition ( Table 3S - Supplementary Material ).

### Moderating effects of positive end-expiratory pressure

A positive interaction of PEEP was found only for the associations of RALE and global LUS score with PaO_2_/FiO_2_ ( Table 4S - Supplementary Material ).

## DISCUSSION

In this study, we investigated the relationship between radiographic and LUS morphological scores of lung edema and several physiological metrics. Our findings suggest that overall, both the RALE score and the global LUS score are poorly correlated with metrics of shunt, dead space, and respiratory mechanics. Counter to our hypothesis, only a few associations improved in patients with ARDS compared to patients without this condition.

The lack of robust associations between radiographic scores and physiological metrics of shunt and dead space is noteworthy. Despite the RALE score and global LUS score potential in diagnosing ARDS,^( 9 , 19 )^monitoring pulmonary aeration and lung edema progression, our results indicate that these morphological scores may not always reliably reflect underlying pathophysiological processes associated with ARDS.^( 19 , 20 )^These findings partially contradict prior research, which demonstrated a strong correlation between LUS scores and aeration measured by computed tomography (CT) in ARDS patients.^( 21 - 23 )^ Yet, our findings align with several other studies. For instance, an investigation involving 37 patients with ARDS revealed a poor correlation between PaO_2_/FiO_2_ and both RALE and LUS scores.^( 24 )^ Another study showed no association between the global LUS score and oxygenation response to prone positioning.^( 25 )^ Furthermore, two additional studies reported no linear relationship between changes in LUS scores and oxygenation metrics.^( 26 , 27 )^ In coronavirus disease 2019 (COVID-19) patients, one study showed no correlation between baseline RALE and oxygenation metrics.^( 12 )^The mechanisms underlying hypoxemia in acute respiratory failure are complex and do not seem to be sufficiently captured by imaging surrogates of lung density.^( 2 )^ Notably, we could not identify previous studies relating dead space surrogates to RALE and global LUS scores; thus, our findings must be seen as hypothesis-generating.

Moreover, the RALE and global LUS scores were originally developed and validated mainly in pulmonary edema and ARDS populations. Their use in patients with alternative etiologies, such as chronic obstructive pulmonary disease exacerbations or drug-induced respiratory depression - which may not exhibit prominent radiographic signs of alveolar-interstitial involvement - may have contributed to the lack of association observed. However, both scores have since also been applied in broader critically ill populations. For instance, the RALE score has been used to characterize radiographic lung edema in general ICU patients undergoing mechanical ventilation.^( 19 , 28 - 30 )^ Lung ultrasound has been used as a diagnostic and monitoring tool in diverse conditions and settings.^( 11 , 31 , 32 )^Therefore, the heterogeneity of the non-ARDS group should be considered when interpreting our findings.

The poor association between morphological scores and compliance^( 33 )^contrasts with findings from a study involving 40 adult ARDS patients, demonstrating a significant and clinically relevant association.^( 14 )^Another study showed how the global LUS score is strongly correlated with CT-measured tissue density, but its changes were not correlated with PEEP-induced recruitment.^( 22 )^A recent study using electrical impedance tomography next to LUS suggested that the LUS score is determined by the ratio between water and gas, compared to water alone.^( 34 )^Albeit unexpected, we did not observe an improvement in several associations among ARDS patients, who typically exhibit greater lung function impairment and imaging abnormalities. While factors such as patient positioning, ventilator settings, and underlying comorbidities may influence the interpretation of radiographic findings and their correlation with physiological metrics,^( 35 )^the heterogeneity of lung injury in ARDS patients may limit the ability of radiographic scores to fully capture the extent of functional impairment.^( 36 )^Conversely, some associations were stronger in patients without ARDS. Our LUS scoring method assigned a score of zero to pleural effusions with underlying C-patterns, following prior studies.^( 16 , 37 )^Nonetheless, this may have underestimated the extent of aeration loss in cases of coexisting consolidation and moderate effusions, potentially contributing to the weak associations observed.

Our study also identified a moderating effect of PEEP on the relationship between radiographic scores and PaO_2_/FiO_2_. This finding underscores the importance of considering ventilator settings when interpreting radiographic assessments in critically ill patients. Adjustments in PEEP levels influence both lung aeration and compliance, thereby impacting the correlation between radiographic scores and physiological metrics.^( 38 )^While the PaO₂/FiO₂ is widely used to assess oxygenation in patients with ARDS, we acknowledge that various factors may influence it. For instance, variations in hemodynamic status, ventilation-perfusion matching, and FiO₂ can significantly alter the PaO₂/FiO₂ independently of underlying changes in pulmonary physiology.^( 39 , 40 )^ Therefore, the PaO₂/FiO₂ remains a practical and globally adopted clinical tool, particularly in ARDS definitions and stratification of hypoxemic respiratory failure.^( 41 , 42 )^ Its use in this study reflects its pragmatic value rather than a strict physiologic equivalence to a shunt.

The strengths of this study rely on the large sample of consecutive patients and the availability of both a radiological and an ultrasound score in the same cohort of patients, with prospective data collection. Several limitations of our study should be acknowledged. To begin, we excluded patients who lacked either baseline LUS or chest X-rays, which may limit the generalizability of our findings by introducing selection bias. This subgroup may differ in clinical severity, potentially impacting external validity. Furthermore, surrogate measures for shunt and dead space may have influenced the observed associations. In addition, the correlation between the two scores found in the current study was lower than previously found in a smaller mixed cohort of non-ARDS and ARDS patients.^( 43 )^ Yet, another study also found no significant association between the RALE score and global LUS score,^( 24 )^in agreement with our findings. Notably, although LUS and chest X-rays were performed within the same calendar day, the exact timing was not always synchronized, which may have introduced variability in the degree of lung aeration captured by the two modalities. Additionally, while the ventilatory ratio is a practical bedside surrogate for dead space ventilation,^( 44 , 45 )^it does not account for CO₂ production variability, which can fluctuate with disease severity, metabolic demand, or nutritional status.^( 46 )^Finally, the study population was drawn from a multicenter cohort in the Netherlands. While this ensures consistency in care standards and imaging protocols, it may limit the external validity of our findings in settings with different ICU practices or patient populations.^( 32 )^

The values observed in our regression models were modest, indicating that imaging scores explained only a small proportion of the variance in physiological parameters. This underscores the multifactorial determinants of gas exchange and respiratory mechanics, including factors beyond structural aeration such as perfusion heterogeneity, inflammatory burden, and cardiac function. Therefore, while imaging scores provide valuable information on lung aeration, their clinical use should be integrated with other functional and hemodynamic assessments. Future studies with prospective designs are warranted to validate our findings and elucidate the complex relationship between radiographic scores and physiological metrics in critically ill patients.

## CONCLUSION

Our study reports the limited association between radiographic morphological scores and metrics of shunt, dead space, and respiratory mechanics in critically ill ventilated patients. A complex moderation effect of acute respiratory distress syndrome status was found, with the association between the radiological score and physiological metrics in some instances being stronger in patients without acute respiratory distress syndrome. These findings underscore the need for a multimodal approach to lung assessment, incorporating both radiographic and physiological metrics to evaluate lung function comprehensively.

Availability of data and materials:

The datasets used and/or analysed during the current study are available from the corresponding author on reasonable request.
