# Supplementary material for: Correlation of sonographic and radiographic scores of lung edema and metrics of shunt, dead space, and respiratory mechanics in invasively ventilated patients
Source: Crit Care Sci. 2025 Sep 25;37:e20250036. doi: 10.62675/2965-2774.20250036 (PMC12614950; doi:10.62675/2965-2774.20250036)
Supplement: Supplementary file 1 [file 2965-2774-ccsci-37-e20250036-suppl01.pdf]

Critical Care Science
https://doi.org/10.62675/2965-2774.20250036

SUPPLEMENTARY MATERIAL

Correlation of sonographic and radiographic scores of lung edema and metrics of shunt, dead space, and respiratory mechanics in invasively ventilated patients

Daan Filippini[1]*, Claudio Zimatore[1]*, Laura A. Hagens[1], Nanon F. L. Heijnen[2], Leila Atmowihardjo[1], Ronny M. Schnabel[2], Dennis C. J. J. Bergmans[2], Daniele Guerino Biasucci[3], Marcus J. Schultz[1], Lieuwe D. J. Bos[1], Marry R. Smit[1], Luigi Pisani[4], on behalf of the DARTS† consortium

*Daan Filippini and Claudio Zimatore are co-first authors
† Diagnosis of Acute Respiratory Distress Syndrome

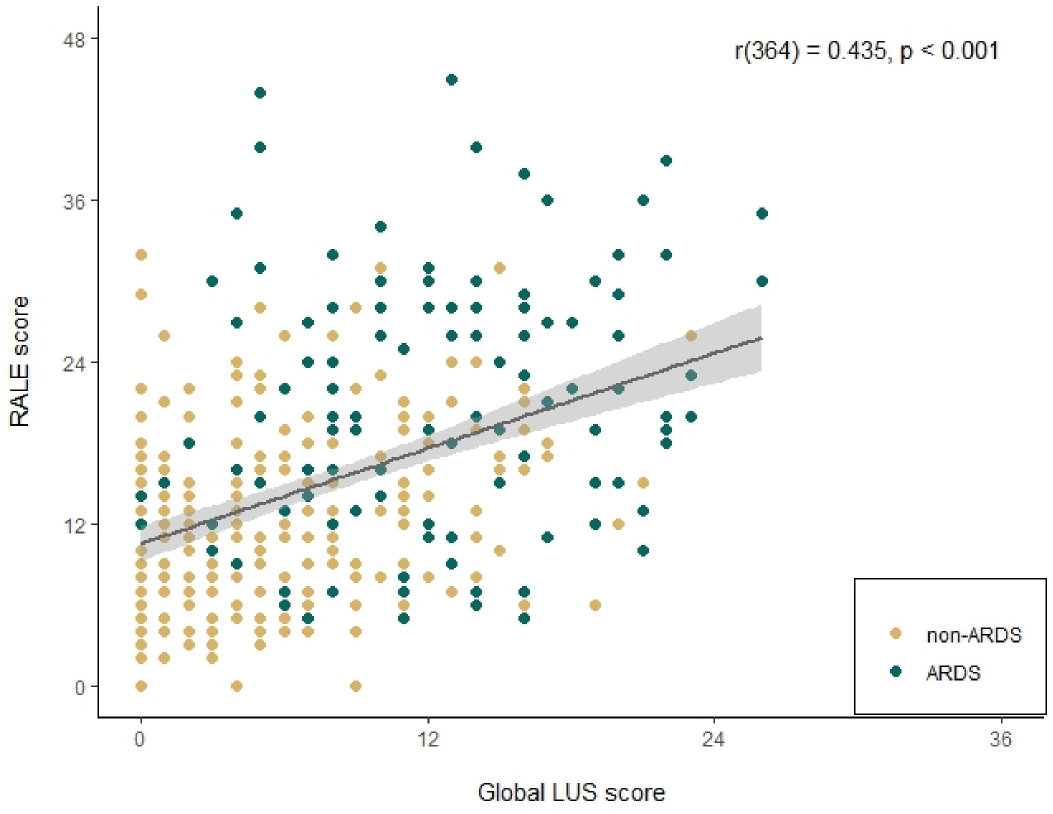

RALE - Radiographic assessment of lung edema; LUS – lung ultrasound; ARDS - acute respiratory distress syndrome.

Figure 1S - Correlation of baseline radiographic assessment of lung edema scores and baseline global lung ultrasound scores.
Individual patient data is displayed as dots. The annotated text represents Pearson correlation coefficients and are displayed as “r(n) = correlation coefficient, p value”.

This is an open access article under the CC BY license (https://creativecommons.org/licenses/by/4.0/).

Table 1S - Linear regression outputs of models containing baseline scores, compared with models containing an interaction with acute respiratory distress syndrome

	RALE score				LUS score		
	β	95%CI	p value		β	95%CI	p value
Shunt							
PaO_2/FiO_2							
Intercept	315	290 - 340	< 0.001	Intercept	323	303 - 342	< 0.001
RALE	-3.1	−4.5 - −1.7	< 0.001	LUS	-6.7	-8.6 - -4.8	< 0.001
Intercept	331	302 - 361	< 0.001	Intercept	334	313 - 356	< 0.001
RALE	-2.4	-4.5- -0.3	0.027	LUS	-5.3	-8.1 - -2.6	< 0.001
ARDS	-151	-209 - -94	< 0.001	ARDS	-100	-148 - -52	< 0.001
RALE × ARDS	3.5	0.5 - 6.5	0.023	LUS × ARDS	2.9	-1.3 - 7.1	0.172
Dead space							
Ventilatory ratio							
Intercept	1.21	1.1 - 1.32	< 0.001	Intercept	1.27	1.18 - 1.36	< 0.001
RALE	0.02	0.01 - 0.02	< 0.001	LUS	0.02	0.01 - 0.03	< 0.001
Intercept	1.3	1.16 - 1.44	< 0.001	Intercept	1.31	1.21 - 1.42	< 0.001
RALE	< 0.01	-0.01 - 0.01	0.545	LUS	< 0.01	-0.01 - 0.02	0.508
ARDS	-0.01	-0.29 - 0.26	0.914	ARDS	0.02	-0.21 - 0.25	0.886
RALE × ARDS	0.01	0 - 0.03	0.058	LUS × ARDS	0.02	0 - 0.04	0.044
Corrected VE							
Intercept	7.3	6.6 - 8.0	< 0.001	Intercept	8.0	7.4 - 8.6	< 0.001
RALE	0.1	0.06 - 0.14	< 0.001	LUS	0.1	0.05 - 0.16	< 0.001
Intercept	7.9	7.0 - 8.8	< 0.001	Intercept	8.3	7.6 - 9.0	< 0.001
RALE	0.03	-0.04 - 0.09	0.431	LUS	-0.02	-0.11 - 0.06	0.609
ARDS	-0.4	-2.2 - 1.3	0.644	ARDS	-0.25	-1.74 - 1.24	0.742
RALE × ARDS	0.09	0 - 0.18	0.050	LUS × ARDS	0.17	0.04 - 0.3	0.009
Mechanics							
Compliance							
Intercept	43.5	39.2 - 47.8	< 0.001	Intercept	43.7	40.1 - 47.2	< 0.001
RALE	-0.28	−0.52 - −0.03	0.027	LUS	-0.5	-0.9 - -0.2	0.002
Intercept	44.2	38.7 - 49.7	< 0.001	Intercept	45.9	41.9 - 50.0	< 0.001
RALE	-0.33	-0.73 - 0.06	0.098	LUS	-0.96	-1.47 - -0.45	< 0.001
ARDS	-2.1	-12.9 - 8.7	0.701	ARDS	-7.9	-17.2 - 1.4	0.094
RALE × ARDS	0.12	-0.44 - 0.68	0.676	LUS × ARDS	0.92	0.13 - 1.72	0.023
Driving pressure							
Intercept	11.7	10.6 - 12.8	< 0.001	Intercept	12.1	11.2 - 13.0	< 0.001
RALE	0.12	0.06 - 0.18	< 0.001	LUS	0.2	0.09 - 0.26	< 0.001
Intercept	12.3	11.0 - 13.8	< 0.001	Intercept	11.6	10.5 - 12.6	< 0.001
RALE	0.05	-0.05 - 0.15	0.300	LUS	0.24	0.11 - 0.38	< 0.001
ARDS	1.4	-4.1 - 1.4	0.325	ARDS	2.27	-0.03 - 4.57	0.053
RALE × ARDS	0.11	-0.03 - 0.25	0.119	LUS × ARDS	-0.19	-0.39 - 0.01	0.062

Continue...

...continuation

Mechanical power							
Intercept	13.3	11.4 - 15.2	< 0.001	Intercept	16.5	14.9 - 18.1	< 0.001
RALE	0.4	0.3 - 0.5	< 0.001	LUS	0.35	0.19 - 0.51	< 0.001
Intercept	15.1	12.7 - 17.4	< 0.001	Intercept	16.3	14.5 - 18.2	< 0.001
RALE	0.18	0.01 - 0.34	0.038	LUS	0.15	-0.09 - 0.38	0.215
ARDS	-1.13	-5.74 - 3.47	0.629	ARDS	4.8	0.7 - 8.8	0.022
RALE × ARDS	0.27	0.03 - 0.51	0.028	LUS × ARDS	0.03	-0.32 - 0.38	0.861

RALE - radiographic assessment of lung edema; LUS - lung ultrasound; 95%CI - 95% confidence interval; PaO_2/FiO_2 - partial pressure of oxygen to fraction of inspired oxygen ratio; ARDS - acute respiratory distress syndrome; VE - minute volume. In bold significant moderation effect by acute respiratory distress syndrome.

Table 2S - Shunt, dead space and respiratory mechanics endpoints and their association with the pulmonary edema scores in the acute respiratory distress syndrome subgroup

	RALE score (ARDS)				LUS score (ARDS)			
	β	95%CI	p value	Adj. R^2	β	95%CI	p value	Adj. R^2
Shunt								
PaO_2/FiO_2								
Intercept	180	139 - 221	< 0.001	0.01	234	198 - 270	< 0.001	0.02
Score	1.15	-0.65 - 2.95	0.208		-2.41	-5.06 - 0.23	0.073	
Dead space								
Ventilatory ratio								
Intercept	1.29	1.02 - 1.55	< 0.001	0.05	1.33	1.1 - 1.56	< 0.001	0.06
Score	0.02	0.01 - 0.03	0.005		0.03	0.01 - 0.04	0.004	
Corrected VE								
Intercept	7.49	5.8 - 9.17	< 0.001	0.06	8.09	6.58 - 9.6	< 0.001	0.05
Score	0.12	0.04 - 0.19	0.002		0.15	0.04 - 0.26	0.008	
Mechanics								
Compliance								
Intercept	42.1	32.3 - 51.9	< 0.001	< 0.01	38.0	29.1 - 46.9	< 0.001	> -0.01
Score	-0.2	-0.6 - 0.2	0.32		-0.04	-0.68 - 0.61	0.912	
Driving pressure								
Intercept	11.0	8.5 - 13.6	< 0.001	0.06	13.9	11.5 - 16.2	< 0.001	> -0.01
Score	0.17	0.05 - 0.28	0.004		0.05	-0.12 - 0.22	0.543	
Mechanical power								
Intercept	13.9	9.8 - 18.1	< 0.001	0.15	21.1	17.1 - 25.1	< 0.001	< 0.01
Score	0.45	0.27 - 0.63	< 0.001		0.18	-0.11 - 0.47	0.226	

RALE - radiographic assessment of lung edema; ARDS - acute respiratory distress syndrome; LUS - lung ultrasound; 95%CI - 95% confidence interval; PaO_2/FiO_2 - partial pressure of oxygen to fraction of inspired oxygen ratio; VE - minute volume.

Table 3S - Shunt, dead space and respiratory mechanics endpoints and their association with the pulmonary edema scores in non-acute respiratory distress syndrome patients

	RALE score (non-ARDS)				LUS score (non-ARDS)			
	β	95%CI	p value	Adj. R^2	β	95%CI	p value	Adj. R^2
Shunt								
PaO_2/FiO_2								
Intercept	331	299 - 363	< 0.001	0.01	334	310 - 358	< 0.001	0.05
Score	-2.36	-4.64 - -0.09	0.042		-5.34	-8.36 - -2.33	0.001	
Dead space								
Ventilatory ratio								
Intercept	1.3	1.17 - 1.43	< 0.001	> -0.01	1.31	1.22 - 1.41	< 0.001	> -0.01
Score	0	-0.01 - 0.01	0.518		0	-0.01 - 0.02	0.48	
Corrected VE								
Intercept	7.9	7.07 - 8.72	< 0.001	> -0.01	8.34	7.72 - 8.96	< 0.001	> -0.01
Score	0.03	-0.03 - 0.08	0.398		-0.02	-0.1 - 0.06	0.582	
Mechanics								
Compliance								
Intercept	44.2	38.9 - 49.6	< 0.001	< 0.01	45.9	42.0 - 49.8	< 0.001	0.06
Score	-0.33	-0.72 - 0.05	0.09		-0.96	-1.45 - -0.46	< 0.001	
Driving pressure								
Intercept	12.4	11.1 - 13.7	< 0.001	< 0.01	11.6	10.6 - 12.5	< 0.001	0.06
Score	0.05	-0.04 - 0.15	0.276		0.24	0.12 - 0.37	< 0.001	
Mechanical power								
Intercept	15.1	12.8 - 17.4	< 0.001	0.02	16.3	14.6 - 18.1	< 0.001	< 0.01
Score	0.18	0.01 - 0.34	0.034		0.15	-0.07 - 0.37	0.192	

RALE - radiographic assessment of lung edema; ARDS - acute respiratory distress syndrome; LUS - lung ultrasound; 95%CI - 95% confidence interval; PaO_2/FiO_2 - partial pressure of oxygen to fraction of inspired oxygen ratio; VE - minute volume.

Table 4S - Linear regression outputs of models containing baseline scores, compared with models containing an interaction with positive end-expiratory pressure

	RALE score				LUS score		
	β	95%CI	p value		β	95%CI	p value
Shunt							
PaO_2/FiO_2				PaO_2/FiO_2			
Intercept	315	290 - 340	< 0.001	Intercept	323	303 - 342	< 0.001
RALE	-3.1	−4.5 - −1.7	< 0.001	LUS	-6.7	-8.6 - -4.8	< 0.001
Intercept	447	384 - 510	< 0.001	Intercept	482	431 - 532	< 0.001
RALE	-5.1	-8.7 - -1.4	0.007	LUS	-14.2	-19.1 - -9.2	< 0.001
PEEP	-20.3	-28.2 - -12.3	< 0.001	PEEP	-21.9	-28.3 - -15.6	< 0.001
RALE × PEEP	0.4	0.04 - 0.8	0.033	LUS × PEEP	1.1	0.5 - 1.6	< 0.001
Dead space							
Ventilatory ratio				Ventilatory ratio			
Intercept	1.21	1.1 - 1.32	< 0.001	Intercept	1.27	1.18 - 1.36	< 0.001
RALE	0.02	0.01 - 0.02	< 0.001	LUS	0.02	0.01 - 0.03	< 0.001
Intercept	1.08	0.78 - 1.38	< 0.001	Intercept	1.15	0.91 - 1.4	< 0.001
RALE	0.01	-0.01 - 0.02	0.433	LUS	< 0.01	-0.02 - 0.03	0.933
PEEP	0.03	-0.01 - 0.06	0.179	PEEP	0.02	-0.01 - 0.05	0.243
RALE × PEEP	< 0.01	< 0.01 - < 0.01	0.633	LUS × PEEP	< 0.01	< 0.01 - < 0.01	0.144
Corrected VE				Corrected VE			
Intercept	7.3	6.6 - 8.0	< 0.001	Intercept	8.0	7.4 - 8.6	< 0.001
RALE	0.1	0.06 - 0.14	< 0.001	LUS	0.1	0.05 - 0.16	< 0.001
Intercept	6.8	4.9 - 8.7	< 0.001	Intercept	6.9	5.3 - 8.5	< 0.001
RALE	0.03	-0.08 - 0.14	0.622	LUS	0	-0.16 - 0.16	0.994
PEEP	0.12	-0.12 - 0.36	0.313	PEEP	0.2	-0.04 - 0.4	0.120
RALE × PEEP	0.01	-0.01 - 0.02	0.399	LUS × PEEP	0.01	-0.01 - 0.03	0.275
Mechanics							
Compliance				Compliance			
Intercept	43.5	39.2 - 47.8	< 0.001	Intercept	43.7	40.1 - 47.2	< 0.001
RALE	-0.28	−0.52 - −0.03	0.027	LUS	-0.5	-0.9 - -0.2	0.002
Intercept	52.8	41.3 - 64.3	< 0.001	Intercept	51.6	42.0 - 61.3	< 0.001
RALE	-0.79	-1.46 - -0.13	0.020	LUS	-1.3	-2.2 - -0.3	0.008
PEEP	-1.23	-2.69 - 0.22	0.097	PEEP	-1.1	-2.3 - 0.2	0.086
RALE × PEEP	0.06	-0.01 - 0.14	0.088	LUS × PEEP	0.09	-0.01 - 0.2	0.089
Driving pressure				Driving pressure			
Intercept	11.7	10.6 - 12.8	< 0.001	Intercept	12.1	11.2 - 13.0	< 0.001
RALE	0.12	0.06 - 0.18	< 0.001	LUS	0.2	0.09 - 0.26	< 0.001
Intercept	9.4	6.5 - 12.4	< 0.001	Intercept	10.4	7.9 - 12.9	< 0.001
RALE	0.18	0.01 - 0.35	0.040	LUS	0.19	-0.05 - 0.43	0.126
PEEP	0.33	-0.04 - 0.71	0.080	PEEP	0.25	-0.07 - 0.56	0.123
RALE × PEEP	-0.01	-0.03 - 0.01	0.314	LUS × PEEP	> 0.01	-0.03 - 0.02	0.780

Continue...

...continuation

Mechanical power				Mechanical power			
Intercept	13.3	11.4 - 15.2	< 0.001	Intercept	16.5	14.9 - 18.1	< 0.001
RALE	0.4	0.3 - 0.5	< 0.001	LUS	0.35	0.19 - 0.51	< 0.001
Intercept	5.6	1.1 - 10.1	0.015	Intercept	6.0	2.1 - 9.8	0.003
RALE	0.18	-0.08 - 0.44	0.176	LUS	0.17	-0.21 - 0.55	0.379
PEEP	1.36	0.79 - 1.93	< 0.001	PEEP	1.52	1.03 - 2.01	< 0.001
RALE × PEEP	< 0.01	-0.03 - 0.03	0.811	LUS × PEEP	< 0.01	-0.04 - 0.05	0.855

RALE - radiographic assessment of lung edema; LUS - lung ultrasound; 95%CI - 95% confidence interval; PaO_2/FiO_2 . partial pressure of oxygen to fraction of inspired oxygen ratio; PEEP - positive end-expiratory pressure; VE - minute volume.
